# A Manual of the Practice of Medicine

**Published:** 1856-07

**Authors:** 


ART. V.—*A Manual of the Practice of Medicine.* By GEORGE HILARO BARLOW, M. A., M. D., Cantab, Fellow of the Royal College of Physicians; Physician to Guy's Hospital; to the Magdalen Hospital and to the Philanthropic Society. With additions by Dr. FRANCIS CONDIE, M. D. Philadelphia: BLANCHARD & LEA. 1856.

Our oft-repeated objections to works in the *practice of medicine*, as distinguished from monographs on special subjects, are still as strong in our mind as ever. The difficulty is insuperable—a compend can never be a treatise—accuracy of description, fullness of detail, careful pathology and discriminating treatment must all yield to the imperious demands of space. Yet it is astonishing to what degree these difficulties are overcome in some few works on general practice. Watson's Practice we have often specified as one replete with sound advice and distinguished by a style of description rarely equalled. So, too, with the book under notice. The vague, careless and wretchedly imperfect pathology and diagnosis, the antiquated treatment which we censured so strongly in Dickson's Practice (criticisms since confirmed by other and higher authority) have here no place. What sins exist are sins of omission — the work, if lacking in detail is free from blunders.

"The treatise before us," says the Medico-Chirurgical Review, "on the important subject of Practical Medicine, bears internal evidence of having proceeded from the pen of an experienced, laborious, and conscientious physician; and we do not hesitate to express our conviction that 'students and junior practitioners,' for whose use it is chiefly intended, may safely adopt it as their guide."

In style — a very essential quality even in a scientific work — Dr. Barlow's Manual is excellent. His manner of description is easy, concise, accurate and readable. In his pathology he is as full as his limits permit, with but few inaccuracies. He refers the cause of rheumatism to uric acid in the blood, therein differing widely from Fuller and from the more general opinion of pathologists, and probably differing also from the fact, for it has been demonstrated by Dr. Garrod, that there is no excess of uric acid in the blood of rheumatic subjects, though it exists very largely in gouty patients, and in them may even be found in the serum of a blister.

Dr. Barlow speaks at some length of the treatment of common colds. His favorite treatment consists of a foot-bath at bed-time, taking also ext. colocynth with one-sixth of a grain of tartar emetic and three grains of ext. hyoscyamus, this to be followed by a saline aperient in the morning, and a diaphoretic mixture of three drachms of liq. ammon acet., half a drachm of spts. æther. nit., and ten or twelve drops of vin. antimonial, in camphor mixture, three or four times daily. Treatment enough for a pneumonia! Another plan is four glasses of sherry, with sugar, in a large quantity of warm water; light reading on the sofa for the evening; and early to bed. There can be little question as to which treatment would be the most popular, or which would do the least mischief. Our own theory is that it is better to wear out a cold than to open the pores and enfeeble the powers of resistance to cold by diaphoretics; and acting on this plan we rarely prescribe anything whatever for a common cold.

In the treatment of pneumonia we find the same English fondness for remedial measures. Referring again to the "Medico-Chirurgical," we quote with approbation the following paragraph from that review: "We think, however, that we should in most cases of pneumonia, abstract less blood and give less mercury than the author appears to recommend. This disease is one of those which, in a large proportion of cases, tend to a spontaneous recovery; and it is unquestionably one of the many which have too often been treated with a mischievous degree of activity."

On the whole our conclusion relative to Barlow's Practice of Medicine, is that if we must have such works this is a good one, the best since Watson, the most accurate in its details, careful in its statements, and sensible in its treatment.

